# Immunoglobulin A nephropathy is characterized by anticommensal humoral immune responses

**DOI:** 10.1172/jci.insight.141289

**Published:** 2022-03-08

**Authors:** Elissa G. Currie, Bryan Coburn, Elisa A. Porfilio, Ping Lam, Olga L. Rojas, Jan Novak, Stuart Yang, Raad B. Chowdhury, Lesley A. Ward, Pauline W. Wang, Khashayar Khaleghi, James An, Sarah Q. Crome, Michelle A. Hladunewich, Sean J. Barbour, Daniel C. Cattran, Rulan S. Parekh, Christoph Licht, Rohan John, Rupert Kaul, Kenneth Croitoru, Scott D. Gray-Owen, David S. Guttman, Jennifer L. Gommerman, Heather N. Reich

**Affiliations:** 1 Department of Molecular Genetics,; 2 Division of Infectious Diseases, Department of Medicine, and; 3 Department of Immunology, University of Toronto, Toronto, Ontario, Canada.; 4 Toronto General Research Institute, University Health Network, Toronto, Ontario, Canada.; 5 Department of Microbiology, University of Alabama at Birmingham, Birmingham Alabama, USA.; 6 Department of Cell & Systems Biology, Centre for the Analysis of Genome Evolution & Function, and; 7 Department of Medicine, University of Toronto, Toronto, Ontario, Canada.; 8 Sunnybrook Health Sciences Centre, Toronto, Ontario, Canada.; 9 Department of Medicine, University of British Columbia, Vancouver, British Columbia, Canada.; 10 Division of Nephrology, University Health Network, Toronto, Ontario, Canada.; 11 Hospital for Sick Children, Toronto, Ontario, Canada.; 12 Department of Laboratory Medicine and Pathology, University of Toronto, Toronto, Ontario, Canada.; 13 Division of Gastroenterology, Mount Sinai Hospital, Toronto, Ontario, Canada.

**Keywords:** Immunology, Nephrology, Chronic kidney disease, Cytokines, Immunoglobulins

## Abstract

IgA nephropathy (IgAN) is a leading cause of kidney failure, yet little is known about the immunopathogenesis of this disease. IgAN is characterized by deposition of IgA in the kidney glomeruli, but the source and stimulus for IgA production are not known. Clinical and experimental data suggest a role for aberrant immune responses to mucosal microbiota in IgAN, and in some countries with high disease prevalence, tonsillectomy is regarded as standard-of-care therapy. To evaluate the relationship between microbiota and mucosal immune responses, we characterized the tonsil microbiota in patients with IgAN versus nonrelated household-matched control group participants and identified increased carriage of the genus *Neisseria* and elevated *Neisseria*-targeted serum IgA in IgAN patients. We reverse-translated these findings in experimental IgAN driven by BAFF overexpression in BAFF-transgenic mice rendered susceptible to *Neisseria* infection by introduction of a humanized CEACAM-1 transgene (B × hC-Tg). Colonization of B × hC-Tg mice with *Neisseria* yielded augmented levels of systemic *Neisseria*-specific IgA. Using a custom ELISPOT assay, we discovered anti-*Neisseria*–specific IgA-secreting cells within the kidneys of these mice. These findings suggest a role for cytokine-driven aberrant mucosal immune responses to oropharyngeal pathobionts, such as *Neisseria,* in the immunopathogenesis of IgAN. Furthermore, in the presence of excess BAFF, pathobiont-specific IgA can be produced in situ within the kidney.

## Introduction

The mechanisms underlying the pathogenesis of IgA nephropathy (IgAN) are poorly understood, yet it is one of the most common causes of kidney failure. The widely accepted “multi-hit hypothesis” for development of IgAN focuses on the production of pathogenic galactose-deficient IgA1-containing immune complexes in the circulation and deposited within kidney glomeruli of patients with IgAN (1). The source (i.e., mucosal-associated lymphoid tissues and/or bone marrow) and stimuli for immune complex production are not known. We have recently demonstrated that IgA-producing plasma cells from the gut can migrate to non-immune system tissues, such as the inflamed brain in the experimental encephalitis model of multiple sclerosis, radically shifting prevailing views on the properties of plasma cells (2). It is therefore tempting to speculate that mucosal-derived IgA-secreting cells could also be recruited to other organs, such as the kidney.

Clinical and experimental evidence suggest a link between IgAN and mucosal microbial exposures. Patients with IgAN experience macroscopic hematuria concurrently with pharyngitis (3), and tonsillectomy is considered standard-of-care for patients with IgAN in Japan (4–6). Our previous studies of a murine IgAN model suggest that commensal microbial colonization is essential for the development of IgAN in mice overexpressing the TNF-family member BAFF (7, 8). This model shares many features with human IgAN, including underglycosylated IgA; clinical and histological measures of kidney injury, such as proteinuria; and IgA-dominant glomerulonephritis (7, 8). The *TNFSF13* (*APRIL*) locus was subsequently linked to IgAN in genome-wide association studies (9). Since APRIL and BAFF share similar receptors, the BAFF/APRIL axis has been implicated in IgAN disease pathogenesis.

We therefore hypothesized that in the setting of high levels of BAFF/APRIL, mucosal-derived IgA-secreting cells are recruited to the kidney and contribute to development of IgAN. To test this hypothesis, we characterized the tonsil and stool microbiome profiles of a large cohort of patients with IgAN compared with household-matched nonrelated control group participants. We identified increased tonsil *Neisseria* carriage and enhanced production of anti-*Neisseria*-targeted IgA in the blood of our patient cohort. We reverse-translated these observations into an experimental IgAN model and found that elevated levels of BAFF provoked an enhanced IgA-biased systemic immune response to mucosal *Neisseria* exposure. We noted increased expression of RNA encoding the secreted-spliced variant of IgA in the kidneys of our experimental mice, suggesting local IgA transcription. Using a custom ELISPOT, we discovered that anti-*Neisseria*–specific IgA secreting cells were detectable in the kidneys of these mice. Our findings showed that a maladaptive host response to a commensal organism was associated with IgAN disease and recruitment of commensal-specific IgA-secreting cells to the kidney.

## Results

### 16s rRNA analysis reveals alterations in the tonsillar microbiome of patients with IgAN compared with healthy controls.

**Figure 1:**
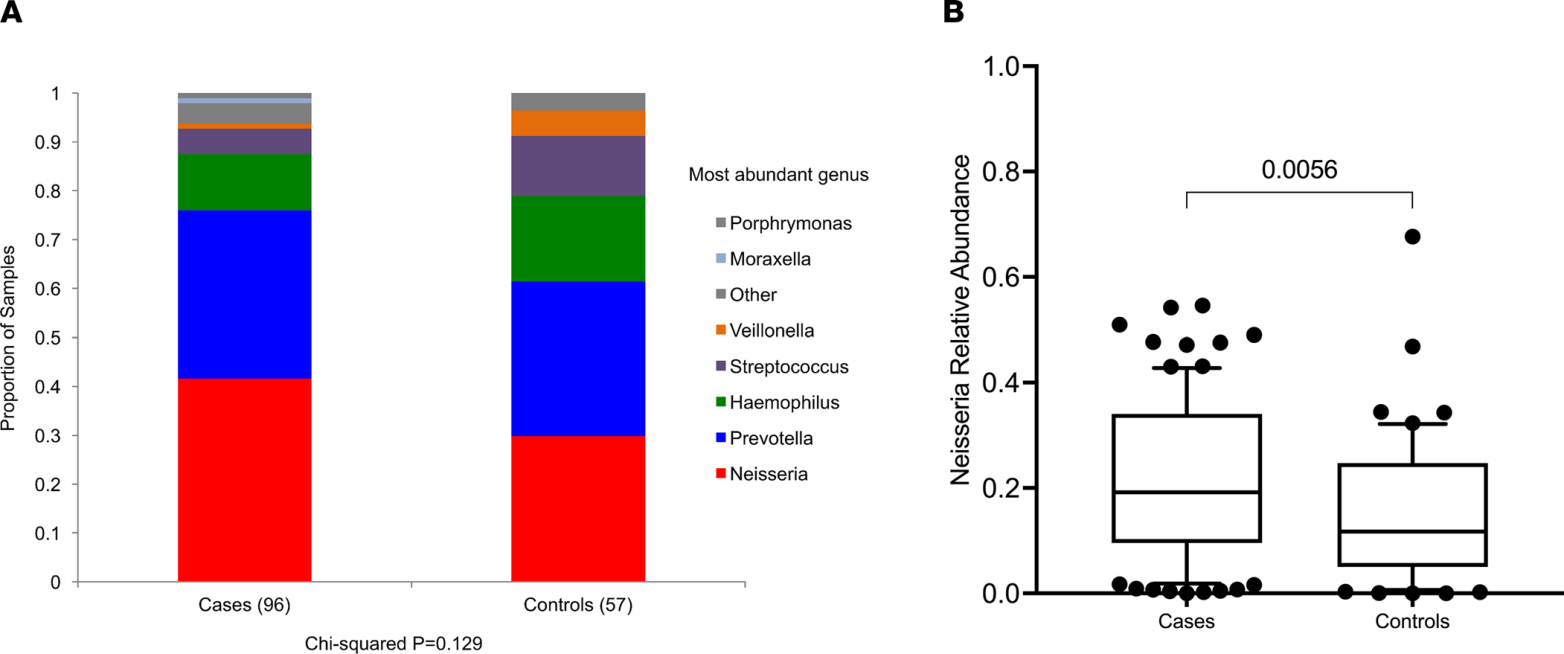
Tonsil microbiota. (**A**) The most abundant genus (>2× the relative abundance of the next most abundant genus) in tonsil swabs in patients with IgAN was *Neisseria*. Each color represents an individual genus, and *y* axis indicates the proportion of samples with that genus as the most abundant organism (χ^2^, *P* = 0.18). (**B**) The relative abundance of *Neisseria* genus was significantly higher in IgAN compared with the nonrelated household-matched healthy control individuals (2-tailed unadjusted *t* test *P* = 0.002).

Given the association between pharyngitis and hematuria in IgAN (4), we compared the tonsillar and stool microbiota of a cohort of patients with IgAN (*n* = 93) with that of healthy nonrelated household-matched individuals (*n* = 58) using 16S rRNA V4 sequencing. Clinical characteristics are provided in Table 1. We did not observe any statistically significant differences in Shannon diversity or taxonomic richness between cases and healthy control tonsil samples (Supplemental Figure 1; supplemental material available online with this article; https://doi.org/10.1172/jci.insight.141289DS1). The dominant tonsillar genus differed between cases and controls (Figure 1A), with a trend toward differences in *Neisseria* as the dominant genus in IgAN tonsil samples (χ^2^ test, *P* = 0.15). We therefore evaluated the difference in relative abundance (RA) of *Neisseria* (Figure 1B) and confirmed the presence of significantly greater abundance of tonsil *Neisseria* in IgAN compared with controls (2-tailed unadjusted *t* test *P* = 0.002). No differences were noted in diversity or taxonomic richness of stool microbiota in IgAN compared with controls (Supplemental Figure 2).

### Humoral immune responses to Neisseria are biased toward IgA versus IgG in patients with IgAN.

**Figure 2:**
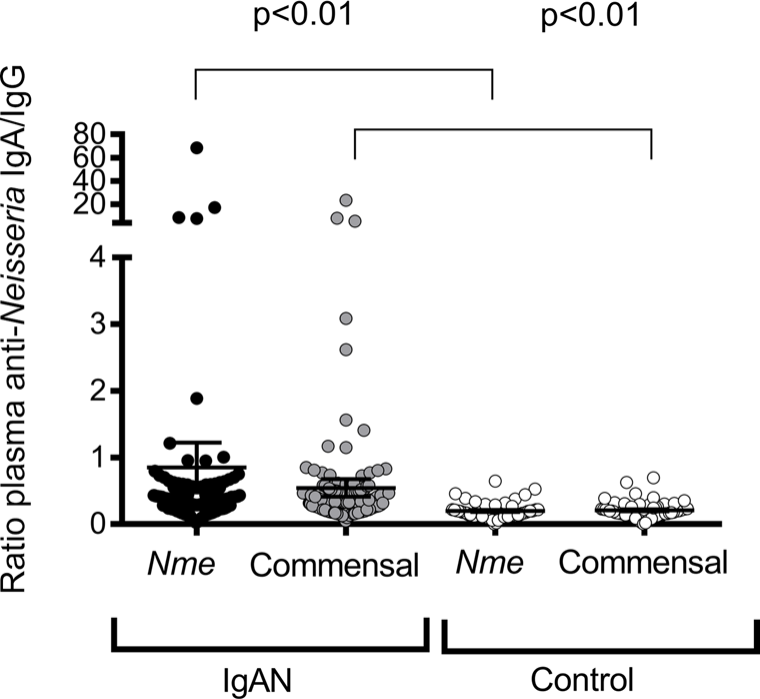
The anti-*Neisseria* response in plasma of patients with IgA nephropathy. Patients with IgAN exhibited exaggerated IgA-biased anti-*Neisseria* responses to both pathogenic and nonpathogenic *Neisseria* species. *Neisseria*-specific antibodies were evaluated across a panel of 4 *N*. *meningitidis (Nme)* strains (90/18311, H4476, 208, and 860800) and 4 commensal strains (*N*. *lactamica*, *N*. *flavescens*, *N*. *sicca, and N*. *cinerea*). Mean and SD shown, Mann-Whitney *U* test, 2-tailed *P* value).

Based on the observed differences in tonsillar microbial abundance, we hypothesized that patients may exhibit altered titers of anticommensal antibodies in their circulation. Because *Neisseria* represented the most abundant genus in patients, we measured anti-*Neisseria* humoral responses, evaluating the ratio of anti-*Neisseria* IgA to anti-*Neisseria* IgG in serum. This ratio is reported as an average IgA/IgG response against a panel of 4 commensal *Neisseria* species (anti–*N*. *lactamica*, *N*. *sicca*, *N*. *cinerea*, and *N*. *flavescens*) and 4 pathogenic *N*. *meningitidis* (*Nme*) strains (90/18311, H4476, 208, and 860800). As illustrated in Figure 2, patients with IgAN exhibited an increase in the ratio of plasma IgA/IgG anti-*Neisseria* antibody titers. Increased IgA titers against *Neisseria* were observed in response to both pathogenic *Nme* and commensal strains (Supplemental Figure 3). Therefore, patients with IgAN exhibited a bias toward generating an enhanced IgA response to a variety of commensal *Neisseria* species and *Nme* strains compared with healthy controls.

### Effect of BAFF overexpression on the immune response to Nme in BAFF-transgenic mice.

We previously reported that the cytokine APRIL (TNFSF13) was elevated in the serum of patients with IgAN compared with controls in 2 independent cohorts (8). We confirmed that APRIL was elevated in patients with IgAN in the current cohort (median 1.98 vs. 1.55; IQR 1.75, 0.38; *P* < 0.01). Moreover, we observed a positive correlation between serum APRIL levels and proteinuria (Spearman’s ρ = 0.28, *P* = 0.01). As in our previous study, we did not observe differences in serum BAFF levels.

APRIL binds to TACI, a TNF receptor that promotes class switch to IgA (10). TACI is also stimulated by higher-order multimers of BAFF, and overexpression of BAFF in BAFF-transgenic (BAFF-Tg) mice is sufficient to stimulate TACI, thus mimicking an APRIL/TACI signal (11). Importantly, as they age, BAFF-Tg mice exhibit an IgAN-like disease characterized by underglycosylated IgA in the serum, IgA deposition in the kidney, elevated proteinuria, and kidney pathology (8). We previously showed that the microbiota is an essential cofactor promoting the deposition of IgA immune complexes in the kidneys of BAFF overexpressing BAFF-Tg mice (8). This implies that a host-microbiome interaction may play a role in the etiology of IgAN, at least in mice. However, in our previous studies, we did not assess whether specific microbial candidates could accelerate the pathogenesis of IgAN-like disease in BAFF-Tg mice.

**Figure 3:**
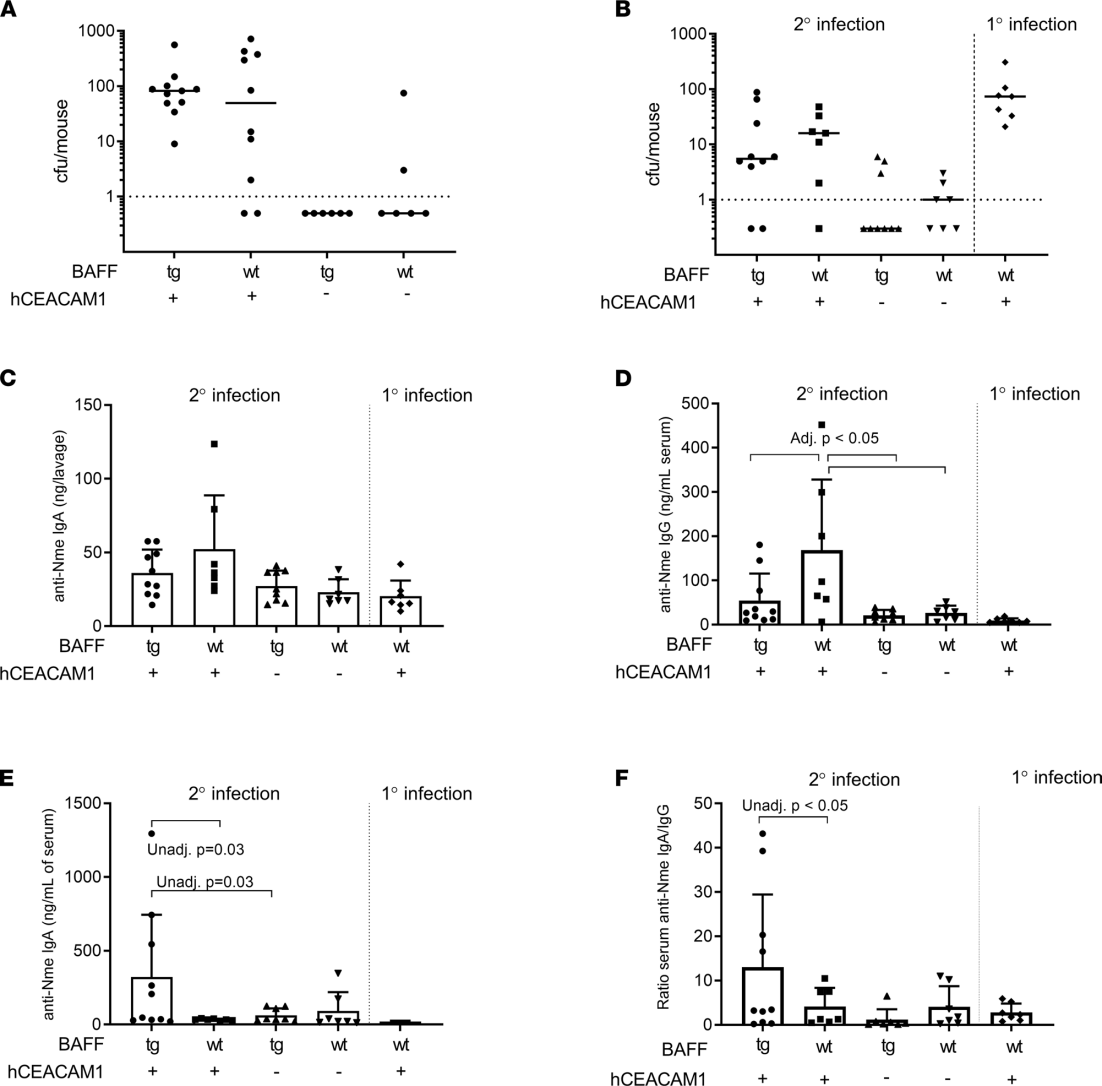
The effect of BAFF overexpression on sterilizing immunity against *N. meningitidis* 90/18311. (**A**) Rate of *Nme* colonization according to genotype during naive infection. (**B**) Bacterial burden 24 hours after secondary infection in littermate controls. Naive hC-Tg mice were used as a comparison for bacterial burden. (**C**) At 24 hours after nasal infection, B × hC-Tg mice did not exhibit an exaggerated anti-*Neisseria* IgA response in the nasopharynx. No anti-*Neisseria* IgG was detected in mice (not shown). (**D**) After nasal infection, hC-Tg mice exhibited enhanced systemic anti-*Nme* IgG response but B × hC-Tg mice did not (1-way ANOVA *F* 5.0, *P* < 0.01, adjusted *P* < 0.05 for indicated comparisons). (**E**) B × hC-Tg mice exhibited an enhanced systemic anti-*Nme* IgA response with 10-fold anti-*Nme* IgA production (1-way ANOVA *F* 2.6, *P* = 0.07). (**F**) Ratio of anti-*Nme* IgA/IgG revealed IgA-biased systemic response in the B × hC-Tg mice (1-way ANOVA *F* 2.3, *P* = 0.09, unadjusted *P* value as shown). All tests 1-way ANOVA with Tukey’s test; adjusted *P* value, mean, and SD shown.

Therefore, we next explored the possibility that *Neisseira*-targeted, IgA-biased immune responses may be observed in BAFF-overexpressing mice. *Nme* infects epithelium via the CEACAM-1 receptor. Because *Nme* will bind to human but not mouse CEACAM-1 we crossed BAFF-Tg mice with mice that express the human form of CEACAM-1 (hC-Tg) to generate double transgenic mice (B × hC-Tg). Transgenic mice and their littermate controls were nasally infected with *Nme*, and serum and nasal lavages were subsequently collected for analysis. As illustrated in Figure 3A, although hC-Tg^neg^ (WT) mice were not appreciably colonized by *Nme*, hC-Tg and B × hC-Tg mice exhibited similar rates of *Nme* colonization (80% vs. 100%, *P* = NS) and similar bacterial burdens (192.9 vs. 116.7, *P* = NS) at 5 days after infection. These data indicate that B × hC-Tg mice did not have altered rates of *Nme* colonization in comparison to WT mice.

We have previously shown that 2 nasal infections are required to induce immunity against a subsequent third nasal infection in hC-Tg mice (12). To determine whether B × hC-Tg mice exhibited improved clearance of *Nme* compared with hC-Tg controls, we nasally infected mice twice, on days 1 and 14. At 24 hours after secondary infection, the rate of infection of hC-Tg mice and B × hC-Tg mice was similar (85% vs. 80%), demonstrating a lack of neutralizing immunity induced by 1 previous exposure of *Nme* (Figure 3B). This implies that overexpression of BAFF did not eliminate the need for 2 infections to induce protection against colonization in our mouse model.

Given that there was no obvious impact of BAFF overexpression on nasal susceptibility to *Nme*, we looked at the mouse antibody response to *Nme* colonization. First, we assessed the local IgA and IgG response to *Nme*. Nasal *Nme-*specific IgG, as evaluated in nasopharyngeal lavage fluid, was not detected in any mice, and nasal anti-*Nme* IgA titers were similar for both B × hC-Tg and hC-Tg mice (Figure 3C). We next measured the systemic antibody response to *Nme* infection. After the second nasal infection, both hC-Tg and B × hC-Tg mice exhibited an *Nme-*specific IgG response in serum above baseline, with hC-Tg mice exhibiting a mean anti-*Nme* IgG titer 2-fold greater than that in B × hC-Tg mice (164 ng/mL vs. 54.6 ng/mL) (Figure 3D). In contrast, B × hC-Tg mice exhibited a mean anti-*Nme* IgA titer in serum that was 10-fold higher than in hC-Tg controls (321.7 ng/mL vs. 33.09 ng/mL) (Figure 3E). Taken together, our findings demonstrated that whereas the local IgA response to *Nme* was comparable between hC-Tg and B × hC-Tg mice, the systemic humoral immune response to nasal *Nme* exposure was IgA-biased in B × hC-Tg mice (Figure 3F).

### Effect of Nme colonization on IgA in the kidneys of BAFF-Tg mice.

**Figure 4:**
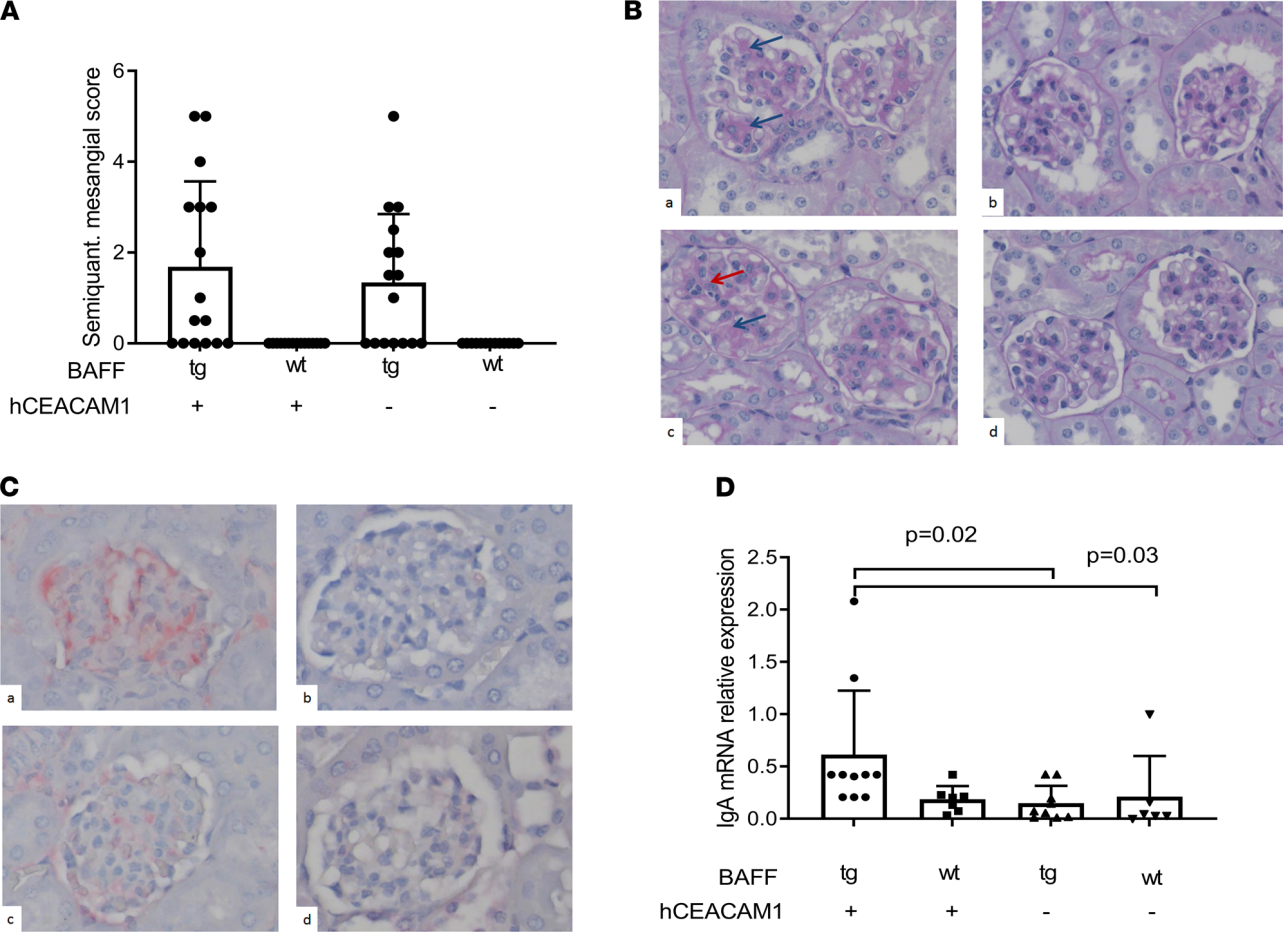
Kidney pathology and IgA expression in experimental IgA nephropathy. (**A**) Semiquantitative scoring revealed mesangial expansion in mice that overexpressed BAFF. (**B**) Representative images corresponding to mesangial matrix scoring (PAS stain, 40× original magnification) in BAFF-Tg × hCEACAM^+^ (a), BAFF-tg × hCEACAM ^+^ (B × hC-Tg) (b), BAFF-wt × hCEACAM^–/–^ (c), and BAFF-wt × hCEACAM1^–/–^ (d). Red arrow shows cellular proliferation; blue arrow shows mesangial expansion. (**C**) Representative sections showing IgA staining by immunohistochemistry (same order as **B**), confirming mesangial deposition of IgA. (**D**) Quantitative evaluation of IgA RNA expression (secreted splice form) in kidney tissue. Kidneys obtained from double transgenic mice (B × hC-Tg) demonstrated the highest degree of IgA mRNA expression (Dunn’s adjusted *P* < 0.05 for comparisons with other groups by Kruskal-Wallis 1-way ANOVA test). Total original magnification, ×40. Mean and SD shown.

Given that systemic *Nme-*specific IgA levels were significantly elevated in BAFF-Tg mice, we hypothesized that a combination of the BAFF transgene and *Nme* infection may accelerate kidney pathology compared with uninfected BAFF-Tg mice. We therefore assessed glomerular mesangial expansion by staining kidney sections with periodic acid–Schiff (PAS). Using a semiquantitative score of mesangial expansion, we observed a nonsignificant trend toward increased mesangial expansion in the B × hC-Tg mice (Figure 4, A and B).

We next examined the impact of *Nme* infection on IgA deposition in the kidney, the diagnostic hallmark of IgAN. Using immunohistochemistry, deposition of mesangial IgG was not detected in any mice (data not shown). Moreover, we did not observe mesangial IgA in the kidneys of WT or hC-Tg mice. In contrast, B × hC-Tg and BAFF-Tg mice exhibited IgA deposition in areas of PAS+ mesangial expansion (representative section in Figure 4C). For a more quantitative analysis, expression of mRNA encoding the secreted splice form of IgA was evaluated by quantitative PCR in the kidneys of all groups of mice (Figure 4D). There was a significant difference in the expression of the secreted splice form of IgA across groups (Kruskal-Wallis 1-way ANOVA, *P* = 0.01), with double transgenic mice (B × hC-Tg) demonstrating significantly higher levels of IgA expression compared with other experimental groups (Dunn’s multiple-comparison test adjusted *P* < 0.05 for all comparisons). These results showed that colonization of BAFF-Tg mice with *Nme* resulted in augmented IgA expression within the kidney itself.

**Figure 5:**
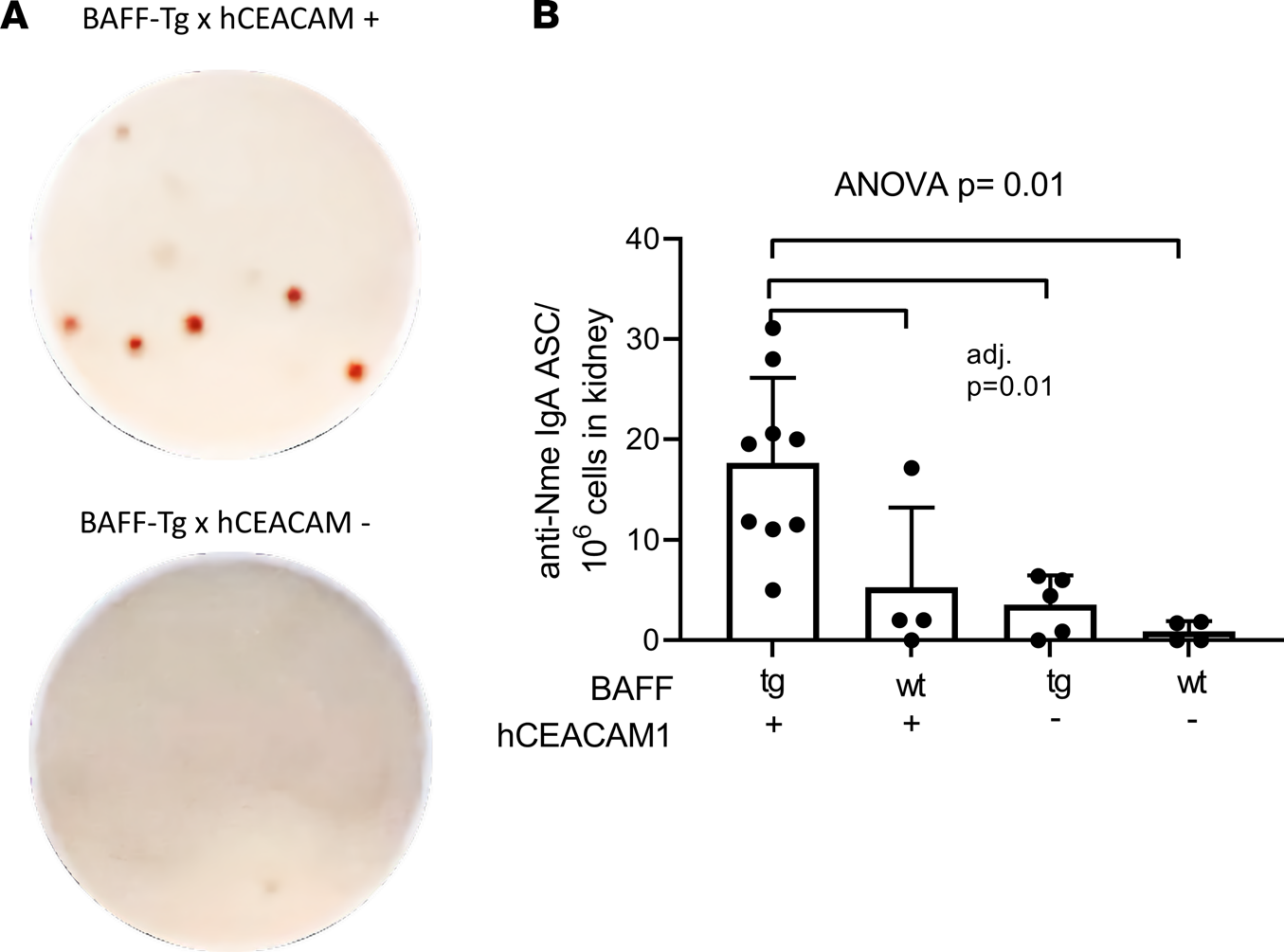
Detection of anti–*N. meningitidis* IgA-secreting cells in the kidneys of BAFF-transgenic mice nasally infected with *N. meningitidis* via hCEACAM-1. (**A**) Representative photos of an ELISPOT assay developed for detection of *Nme*-reactive IgA-producing cells. (**B**) Anti-*Nme*-specific IgA–antibody-secreting cells (igA-ACS) were detected predominantly in kidneys of BAFF-Tg × hCEACAM^+^ (B × hC-Tg) mice (mean and SD shown, 1-way ANOVA with Tukey’s test, adjusted *P* = 0.01 for all comparisons indicated).

Having recently discovered that mucosal-derived IgA-secreting cells migrate to brain tissue in mice with experimental encephalitis (2), we hypothesized that the intrarenal expression of IgA mRNA may reflect the presence of anticommensal IgA-producing cells that have migrated from the site of initial pathobiont exposure. We therefore designed a custom ELISPOT assay to detect *Nme*-specific antibody-secreting cells. High-affinity ELISPOT plates were coated with heat-killed *Nme* and incubated with a single-cell suspension of kidney-derived immune cells from our experimental mice; HRP-conjugated IgA detection antibodies were subsequently added. We discovered that *Nme*-specific IgA-secreting cells were identified in the kidney parenchyma of *Nme*-infected mice, predominantly in the setting of increased BAFF levels (B × hC-Tg mice, Figure 5).

## Discussion

The diagnostic hallmark of IgAN is the deposition of nephritogenic galactose-deficient IgA-containing immune complexes in the glomerular mesangium; however, the origin of and trigger for pathogenic Gd-IgA1 are not known. Genome-wide association studies have demonstrated a *TNFSF13* (*APRIL*) variant is associated with IgAN susceptibility (9) and contributes to IgAN pathology in an experimental model, and elevated APRIL levels are documented in patients with IgAN. Our work suggests that in the setting of high levels of BAFF/APRIL, pathobiont-specific IgA is produced in situ within the kidney by mucosal-derived antibody-secreting cells that may contribute to development of IgAN.

Given the clinical and experimental association between mucosal microbial exposure and IgAN, we initially hypothesized that there would be differences in commensal mucosal microbiota in patients with IgAN. We observed significantly higher rates of tonsil colonization with *Neisseria* in a large cohort of patients with IgAN compared with healthy nonrelated household-matched controls. Moreover, patients with IgAN demonstrated increased levels of serum IgA specifically targeted against both commensal and pathogenic *Neisseria* species.

We speculated that an augmented IgA response to *Neisseria* may contribute to IgAN pathogenesis. While it is unlikely that *Neisseria* species are the only microbial trigger to contribute to IgAN pathogenesis, this finding served as a valuable opportunity to explore how a humoral immune response to a mucosal pathobiont may contribute to the immunopathogenesis of IgAN. We turned to our experimental IgAN model where we previously demonstrated that BAFF overexpression promotes an IgAN-type kidney disease that is dependent on the presence of commensal microbiota (8).

Given the requirement of the human CEACAM receptor to colonize our B-Tg mice with *Neisseria*, our model provided an opportunity to evaluate the impact of colonization by a selected organism on mucosal immune responses and antibody-secreting cell migration. Our findings in this model confirmed our observations in humans that the host response to an oropharyngeal pathobiont, such as *Neisseria,* is associated with aberrant accumulation of IgA producing cells in the kidney and that the BAFF/APRIL axis is a key cofactor in this response.

We did not observe significant differences in the renal histopathology after *Nme* infection, likely due to the limitations of our model. Generation of marked differences in renal pathology would likely require more time. However, the time frame that we could maintain *Nme* colonization safely without compromising the health of the mice was relatively short and this limited our ability to assess long-term kidney injury.

There is emerging evidence that IgA-producing cells reactive against antigens present at mucosal surfaces can be found beyond mucosal-associated lymphoid tissues. For example, commensal-targeted IgA-producing plasma cells can be detected in the blood and bone marrow (13). We have recently discovered that IgA-producing plasma cells and/or plasmablasts egress from the gut and migrate to the CNS in response to induction of experimental encephalitis (2). Our current work suggests that IgA-producing cells induced by *Nme* exposure in the airways migrate to the kidney. A recent paper suggests the presence of CD19-positive B cells that colocalize with IgA in human IgAN biopsies (14). Further work is required to determine the phenotype of the anti-*Nme* IgA-producing cells localized in the kidney and the mechanisms responsible for facilitating egress of cells to remote sites.

The relative contribution of the tonsillar versus gut-associated lymphoid tissue to the development of IgAN is a topic of ongoing debate in the nephrology community and has implications for clinical care (15, 16). Tonsillectomy is routinely performed to treat IgAN in Japan, supported by clinical trials demonstrating efficacy (4, 17). Production of APRIL within tonsillectomy samples is enhanced in patients with IgAN compared with non-IgAN samples (18).

Given the important role of gut-associated lymphoid tissue in IgA production, it is surprising that we did not observe differences in stool microbiota in patients with IgAN, particularly since this finding is in contrast to previous reports (19). There are several possible explanations. Our study may not have been adequately powered to detect these differences. As demonstrated in inflammatory bowel disease, a more sensitive approach may be required, such as targeted sequencing of IgA-coated bacteria, to identify disease-causing species (20). It is also possible that in mice or in individuals that hyper-produce IgA, the transport of IgA into the lumen via the polymeric IgA receptor may be a rate-limiting step, and saturation of this transport mechanism could result in a limited or negligible effect on the microbiome. Despite these potential limitations, it is tempting to speculate that our data support the possibility that tonsil microbial exposures play a more dominant role in production of nephritogenic IgA.

Despite the longstanding suspected relationship between microbial exposures and IgAN, studies of systemic microbe-specific IgA responses are limited. Our study demonstrated that IgA responses to an upper airway commensal microbe were augmented in IgAN. Our mouse model demonstrated that the BAFF/APRIL axis can provoke potentially maladaptive IgA responses to upper airway bacterial challenges. Moreover, organism-specific IgA-producing cells were detectable within the kidney tissue. Thus, in the case of genetically susceptible individuals, a normally harmless commensal organism may assume the role of a pathobiont insofar as it has the ability, in concert with the host immune system, to provoke a pathogenic response that causes IgA-dominant kidney disease.

## Methods

### Clinical cohort.

We recruited 93 individuals with biopsy-proven IgAN and 58 primarily household-matched nonrelated healthy control volunteers. Adult patients with biopsy-proven IgAN were eligible for study if they had no history of concurrent systemic illness or infection and had an estimated glomerular filtration rate (eGFR) greater than 30 mL/min/1.73 m^2^. Control group participants had no history of kidney disease or systemic illness. Whenever possible, this group consisted of non-genetically-related household-matched individuals to minimize differences in dietary habits and environment. Study participants could not have received antibiotics within 6 months of microbiota sampling.

The characteristics of patients enrolled in the study are provided in Supplemental Table 1. Patients enrolled in the study had a spectrum of disease severity and were comparable with controls with respect to age and self-reported race. Patients had relatively preserved renal function, with eGFR > 30 mL/min/1.73 m^2^.

### Microbiota characterization.

Tonsil bacteria were sampled using 2 sterile specimen collection swabs, which were placed immediately into cryovials and stored at –80°C. The samples were suspended in PBS and proteinase K and disrupted by vortex. Bacterial DNA was extracted using the DNeasy Blood and Tissue column-based kit (Qiagen). The DNA integrity and quantity were evaluated using a Nanodrop spectrophotometer (Thermo Fisher Scientific). Individuals were instructed to collect their stool using a standard sterile specimen collection container and then to place it in the home freezer. All stool samples were snap-frozen upon receipt at the research lab. After sample homogenization with 0.1 mm glass beads (MoBio), stool bacterial DNA was extracted using the QIAamp DNA Stool Mini kit (Qiagen) according to the manufacturer’s protocol.

The V4 hypervariable region of the 16S rRNA gene was amplified using a universal forward sequencing primer and a uniquely barcoded reverse sequencing primer to allow for multiplexing (21). Amplification reactions were performed using KAPA2G Robust HotStart ReadyMix (KAPA Biosystems) following the manufacturer’s protocol. For saliva samples, the V4 region was amplified by an initial denaturation at 95°C for 3 minutes, followed by 24 cycles of 95°C for 15 seconds, 50°C for 15 seconds, and 72°C for 15 seconds, ending with a 5-minute extension at 72°C. For stool samples, the amplification reactions were carried out for 17 cycles instead of 22 cycles. All amplification reactions were performed in triplicate, checked on a 1% agarose tris-borate-EDTA electrophoretic gel (TBE) gel, and then pooled at even concentrations. The final library was purified using Agencourt AMPure XP beads (Beckman Coulter). The purified library was quantified and sequenced on the Illumina MiSeq System, according to the manufacturer’s instructions, using the 300 cycle V2 sequencing chemistry to generate 150 × 2 paired-end reads.

Sequencing data were demultiplexed using QIIME2 standard paired-end demultiplexing protocol or directly on the Illumina MiSeq. The UNOISE pipeline, available through USEARCH version 10.0.240, was used for sequence analysis (22–24). The last base, typically error-prone, was removed from all the sequences. Sequences were assembled and quality-trimmed using –fastq_mergepairs and –fastq_filter, with a –fastq_maxee set at 1.0 and 0.5, respectively. Assembled sequences less than 233 bp were removed. Following the UNOISE pipeline, unique sequences were identified from the merged pairs and sorted. Sequences were denoised and chimeras were removed using the unoise3 command in USEARCH. Assembled sequences were then mapped back to the chimera-free denoised sequences at 97% identity operational taxonomic units (OTUs) using the –usearch_global command. Taxonomy assignment was executed using SINTAX (25), available through USEARCH, and the SINTAX compatible Ribosomal Database Project (RDP) database version 16, with the default minimum confidence cutoff of 0.8 (26). OTU sequences were aligned using PyNast accessed through QIIME (27). Sequences that did not align were removed from the data set, and a phylogenetic tree of the filtered aligned sequence data was made using FastTree (28). Low-abundance OTUs (<0.005% RA) were removed from the OTU table (29).

Beta diversity was calculated using QIIME (27). The data were rarefied to an even depth of 10,000 sequences per sample. Principal coordinate analysis plots were of the rarefied data using QIIME and plotted using EMPeror (30).

### Preparation of Neisseria cultures.

*Neisseria* strains were cultured on gonococcus (GC) agar supplemented with IsoVitalex and in the context of mouse experiments, VCNT (vancomycin, colistin, nystatin, and trimethoprim) inhibitor (Becton Dickinson) at 37°C with 5% CO_2_. Log phase growth was achieved by transferring overnight cultures from GC agar into 10 mL of brain heart infusion (BHI) broth (Becton Dickinson) supplemented with 1% IsoVitalex and incubated at 37°C with agitation for 4 hours. Broth cultures were diluted to an OD_600_ = 0.2 prior to heat-killing at 65°C for 30 minutes.

### Serum procedures.

To measure APRIL in serum samples, we used a commercially available kit (eBioscience, BMS2008). The kit protocol was used with the following modifications: human serum samples were diluted 1:3 in nuclease-free water and subsequently heat-inactivated in a 60°C water bath for 5 minutes. Heat-inactivated samples were then loaded neat onto the microwell strips without using sample diluent. Standard was diluted in the plate, in duplicate, as indicated in the protocol but extended for a total of 13 dilutions followed by a sample diluent blank, a water blank, and an internal serum control sample from a female study participant without IgAN, heat-inactivated in the same manner as the other samples. Samples were incubated without the anti-APRIL detection antibody for 2 hours, as per the protocol. After washing the plates, 50 μL/well of the anti–APRIL-biotin antibody was added for 1 hour and shaken at room temperature, as per the protocol. Lastly, the plates were developed for 12–13 minutes before stopping the reaction and reading on a photospectrometer at 450 nm.

Serum *Neisseria*-specific IgA and IgG were measured by a custom whole-bacteria ELISA as previously described (12). Briefly, multi-well plates (Nunc Maxisorp, Nalgene) were coated with heat-killed *Neisseria* strains and left to dry overnight, and then washed and blocked with 5% BSA. Patient plasma samples were diluted 1:1000 and assayed for *Neisseria*-specific antibodies across a panel of 4 *Nme* strains (90/18311, H4476, 208, and 860800) and 4 commensal *Neisseria* strains (*N*. *lactamica*, *N*. *flavescens*, *N*. *sicca, and N*. *cinerea*). Patient samples and standards were applied to the wells in duplicate and incubated overnight. A standard curve was created using human plasma IgA (Calbiochem). After incubation, plates were washed again and the detection antibody, an alkaline phosphatase–conjugated (AP-conjugated) goat antibody targeting human IgA (Jackson ImmunoResearch), was applied. Absorbance at 600 nm wavelength was measured after 40-minute incubation with BluePhos AP detection substrate (SeraCare, KPL).

### Mice.

BAFF-Tg mice (obtained from Ann Ranger and Jeff Browning, Biogen Inc.) were backcrossed with WT mice (Charles River Laboratories) and subsequently interbred as BAFF-Tg^+/+^ or BAFF-Tg^+/–^ mice (7, 8). Mice that express transgenic human CEACAM-1 (hC-Tg) have been previously described (12). These mice were crossed with BAFF-Tg mice to generate BAFF × hCEACAM-Tg progeny (B × hC-Tg) for infection experiments.

### Tissue mRNA expression.

At the time of euthanization, a section of kidney tissue was preserved in formalin and paraffin-embedded to be used for morphological assessments and RNA extraction. Tissue was sectioned and collected in 100% xylene for deparaffinization and washed with 75% ethanol. Tissue disruption and protease digestion were performed as previously described (31). The Recoverall kit (Invitrogen) was used for remaining steps of column-based RNA extraction. Reverse transcription was performed using cDNA generated using SuperScript IV Reverse Transcriptase (Invitrogen). Abundance of the secreted-spliced variant of IgA mRNA was quantitated using real-time PCR with custom primers and normalized to GAPDH expression. IgA: forward 5′-GCC TTG CCC ATG AAC TTC AC-3′, reverse 5′-CGC TGA CAT TGG TGG GTT TA-3′; GAPDH: forward 5′-CAT GGC CTT CCG TGT TCC TA-3′, reverse 5′-GCG GCA CGT AG ATC CA-3′. Samples were diluted with RNase free water and reactions contained 5 μL 2× Maxima SYBR Green (Thermo Fisher Scientific), 0.4 μL each of forward and reverse primers, 2.2 μL nuclease-free water, and 2 μL cDNA. All reactions were run in duplicate on a 384-well plate using the Bio-Rad CFX384 Touch Real-Time PCR Detection System.

### In vivo Neisseria infections.

WT (hC-Tg^neg^), C-Tg, and littermate B × hC-Tg mice were nasally infected with 1 × 10^5^ CFUs of *Nme* strain 90/18311 resuspended in 10 μL of PBS. Mice were infected at day 1 (primary infection) and, where indicated, day 14 (secondary infection). Mice were euthanized via CO_2_ inhalation 5 days after primary infection or 1–4 days following the secondary infection. Serum used to measure systemic responses to *Nme* was collected via cardiac puncture; mucosal samples were obtained via tracheal/nasal lavages. Bacterial burden per mouse was enumerated by swabbing nasal cavities and plating resuspended fluid overnight at 37°C.

### Commensal-specific ELISPOT.

Membrane plates (0.45 μm Hydrophobic High Protein Binding Immobilon-P Membrane, MilliporeSigma) were coated with 5 mg/mL of heat-killed inactivated *Nme* and placed at 4°C overnight. The plates were blocked the next day with 10% FBS/complete RPMI for at least 2 hours at 37°C. Single-cell suspensions were loaded onto the plate at serial 2-fold dilutions in 10% FBS/complete RPMI and incubated overnight at 37°C. Cells were removed the next day and washed with 0.1% Tween 20/PBS 5×. HRP-conjugated IgA and AP-conjugated IgG (in the case of 2-color ELISPOT) detection antibodies were subsequently added for 2 hours at 37°C.

Plates were then washed with 0.1% Tween 20/PBS 3× and with PBS 3×. The plates were developed while covered with aluminum foil until spots were visible using 3-amino-9-ethylcarbazole (AEC) peroxidase (for HRP-conjugated antibodies, Vector Laboratories) and Vector blue (for AP-conjugated antibodies, Vector Laboratories) substrates. After the development was done, the plates were washed with distilled water and were left to dry overnight. The spots were counted based on the original cell dilution.

### Preparation of single-cell suspensions from kidney.

Mouse kidneys were collected in 10% FBS/complete RPMI and kept on ice until digestion was performed. The kidneys were perfused with PBS for the ELISPOT tests. Multi Tissue Dissociation Kit 1 enzymes (Miltenyi Biotec) were prewarmed to 37°C and in accordance with the manufacturer’s recommendations. Both mouse kidneys were incubated with enzymes in a gentleMACS C tube using MACSmix Tube Rotator for 10 minutes at 37°C in a CO_2_ incubator. Next, the mixture was briefly transferred to a 6-well plate and mechanically dissociated with tweezers into approximately 1 mm × 1 mm pieces, and then returned to the C tube for an additional 10 minutes of incubation as above. After incubation, a gentle MACS dissociator was used for a final mechanical digestion step prior to filtering through 70 μm nylon mesh and inactivation of enzymes using ice-cold RPMI with 10% FBS.

A Percoll gradient (30% Percoll for 20 minutes at 200 rpm [730 relative centrifugal force × *g*]) was performed on the cells obtained from the digested kidneys to enrich for immune cells. The pellet was then resuspended in 1 mL of RBC lysis buffer (155 mM NH_4_Cl, 12 mM NaHCO_3_, 0.1 mM EDTA) for 5 minutes on ice. The cells were washed with PBS 1× and with 10% FBS/complete RPMI 1× and were then ready to be plated.

### Statistics.

Data distribution was evaluated and group comparisons were performed using unpaired parametric or nonparametric tests as appropriate. A *P* value less than 0.05 was considered statistically significant.

### Study approval.

All clinical investigation was conducted according to Declaration of Helsinki principles. The study received approval from the research ethics boards of University Health Network and Sunnybrook (Toronto), University of Toronto, and University of British Columbia. The clinical study was approved by the research ethics boards of all clinical centers (coordinating center UHN REB 11-0748). All animal experiments were conducted with ethical approval from the University of Toronto, Faculty of Medicine animal care committee (protocols 2001 1363, 1365). Written informed consent was obtained from all study subjects.

## Author contributions

PL, JN, RBC, MAH, SJB, DCC, RSP, CL, KC, RJ, JLG, and HNR contributed to patient study design, enrollment, and clinical characterization. BC, PWW, RK, KC, and DSG contributed to microbiota study design and execution, and data analyses. EGC, EAP, OLR, JN, SY, LAW, KK, JA, SQC, SDGO, JLG, and HNR performed design, execution, and analyses of human APRIL, antibody, and murine experimental data. RJ designed the nephropathology experiments, generated histologic scores, and contributed to pathology data interpretation. All authors contributed to manuscript drafting and critical review. JLG and HNR contributed equally. The order of co–senior authors was decided based on the timeline of their contributions.

## Supplementary Material

Supplemental data

## Figures and Tables

**Table 1:**
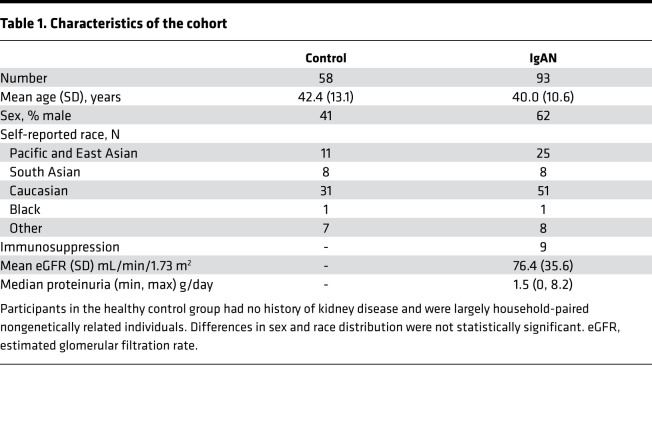
Characteristics of the cohort

## Version 1 02/08/2022

In-Press Preview

## Version 2 03/08/2022

Electronic publication
