# Supplementary material for: Immunoglobulin A nephropathy is characterized by anticommensal humoral immune responses
Source: JCI Insight. 2022 Mar 8;7(5):e141289. doi: 10.1172/jci.insight.141289 (PMC8983137; doi:10.1172/jci.insight.141289)

Supplementary Figures

Supplementary Figure 1.
Alpha-diversity of tonsillar microbiota (rarefied to 5826 sequences/sample) was compared between cases and controls (A). Neither Shannon Diversity nor observed operational taxonomic units (OTUs) differed between cases and controls (B). Goods coverage estimator demonstrated adequate sampling depth at 5826 sequences/sample (C). There were no differences in sample ordination between cases (red) or controls (blue) by principal coordinates analysis of Bray-Curtis dissimilarity (panel D). Principle components analysis did not reveal distinct clustering of cases and controls.

A.

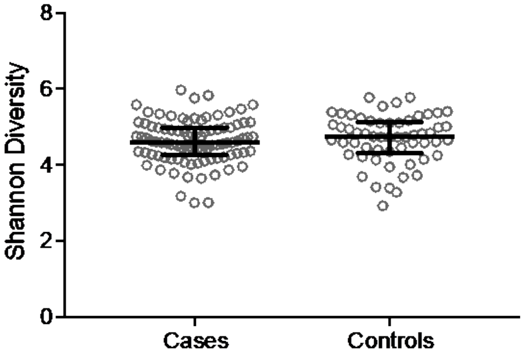

B.

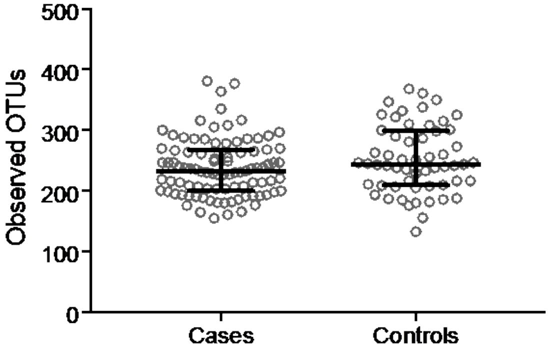

C.

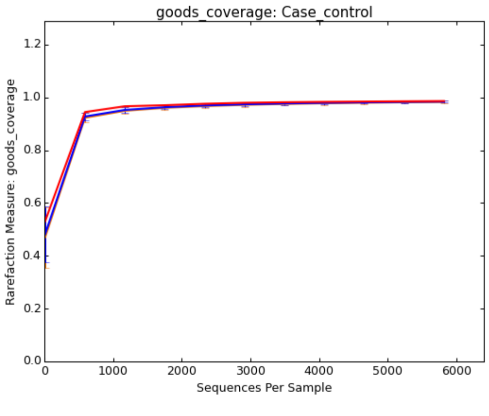

D.

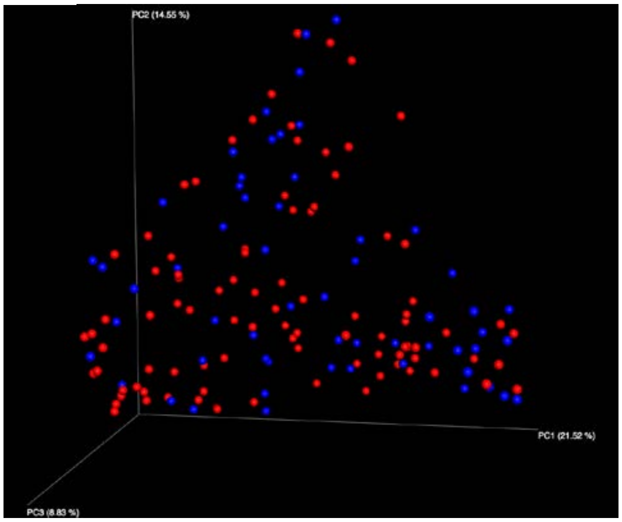

Supplementary Figure 2. Stool analysis in 139 human subjects and in mice.

A. Principal components analysis plots of stool samples. There is no clear separation of groups based upon stool microbiome. Plots are coloured according to sample type: IgAN (red) and healthy control (blue). Panel a. Bray-Curtis distances Panel b. Weighted UniFrac distances.

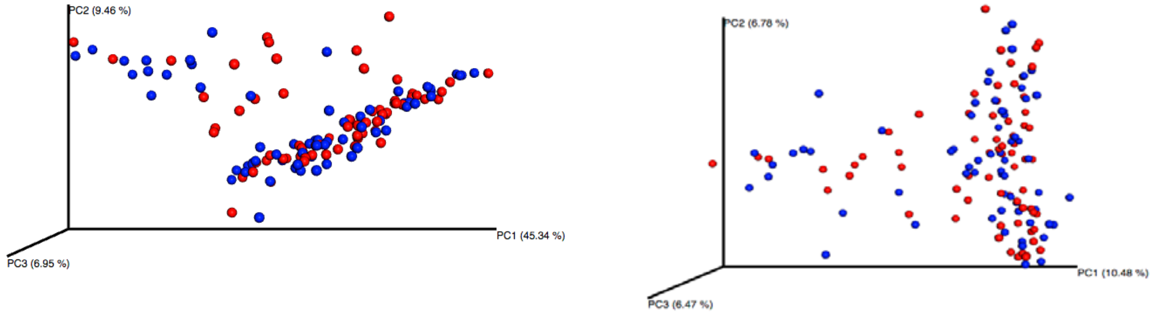

B. Supplementary Figure 2. Stool analysis in experimental model. Bray-Curtis principal coordinates analysis of stool microbiota in BAFF transgenic (blue) versus wild-type (red) mice (A) and mice from two parental lineages (dams 1 and 2, panel B) demonstrating that lineage exerts a strong effect on stool microbiota compared to presence/absence of the transgene. Axes represent the degree of variance explained by each axis as indicated.

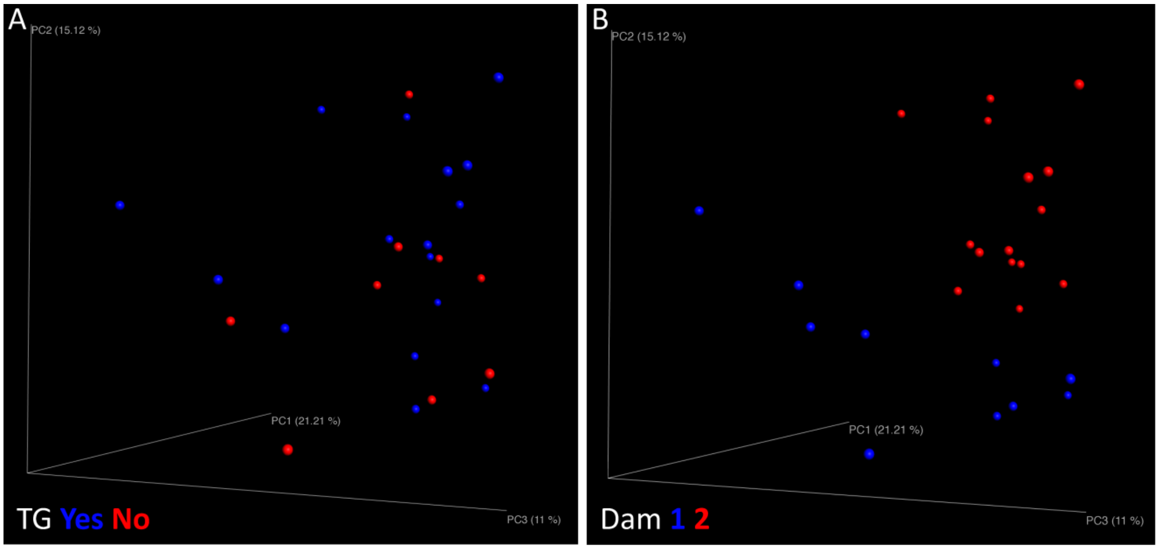

Supplementary Figure 3.

The *Neisseria*-specific IgA/IgG ratio. Pathogenic *N. meningitidis* strains in order of columns: 90183112, h44763, 208, 8608004. Commensal species in order of columns: *N. flavescens, N. sicca, N. cinerea, N. Lactamica*.

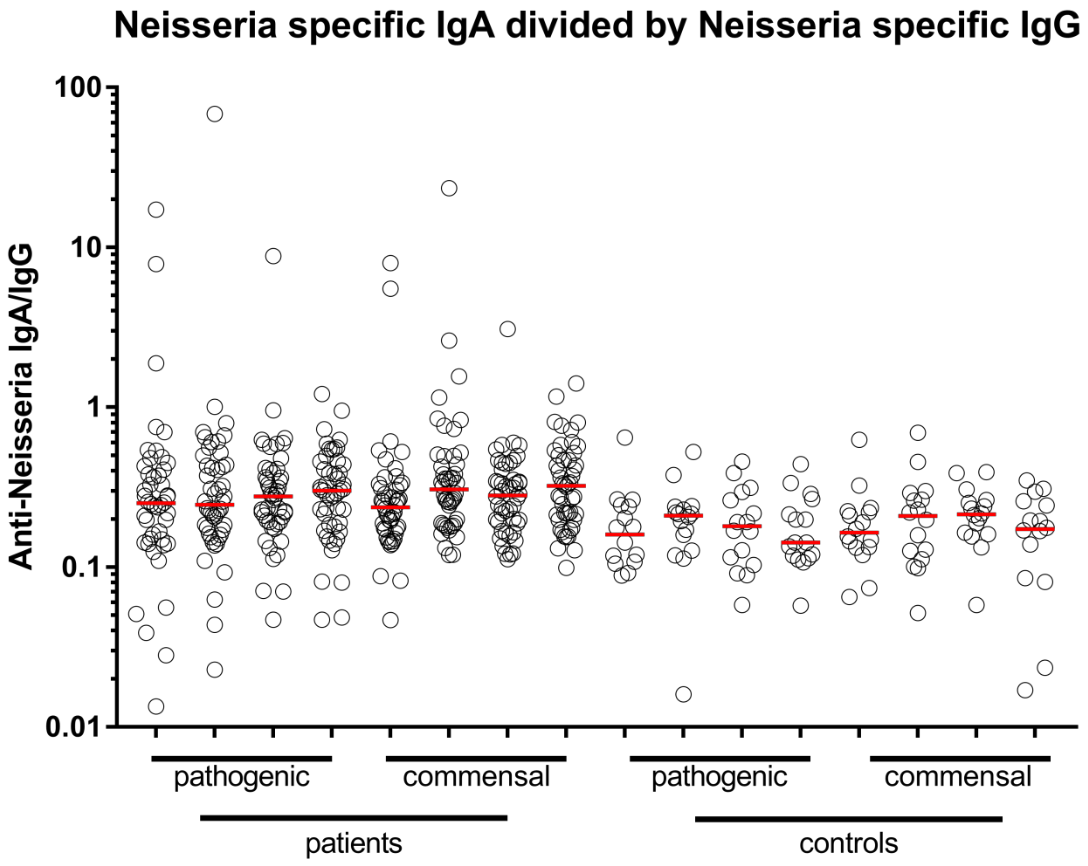

Section 2: Breeding Schemes

Husbandry design for microbiome and infection analyses.

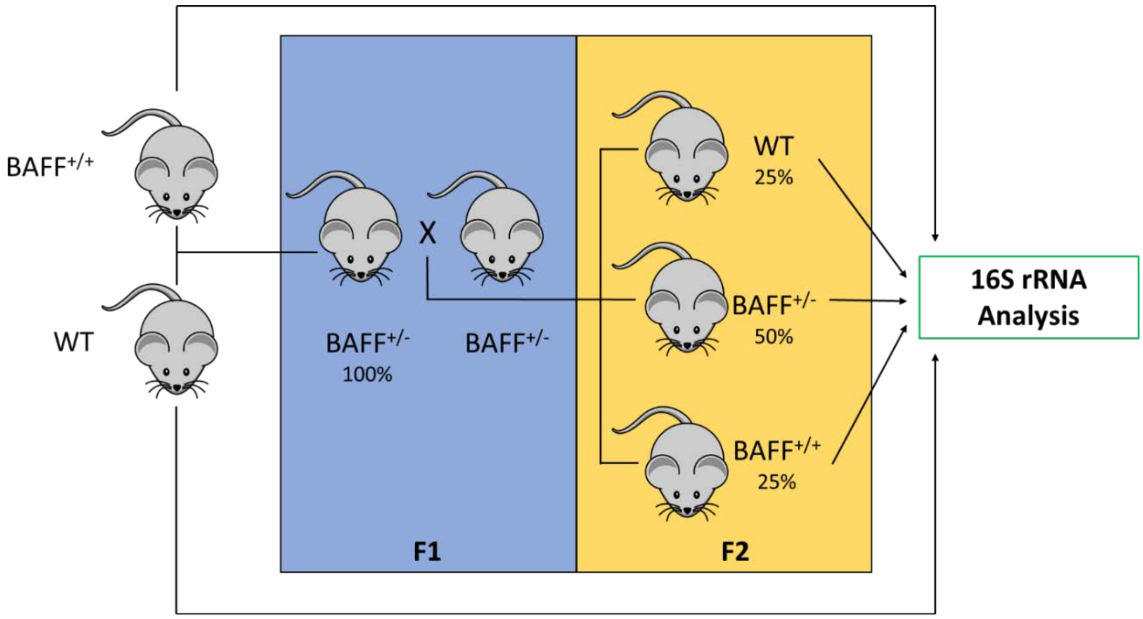

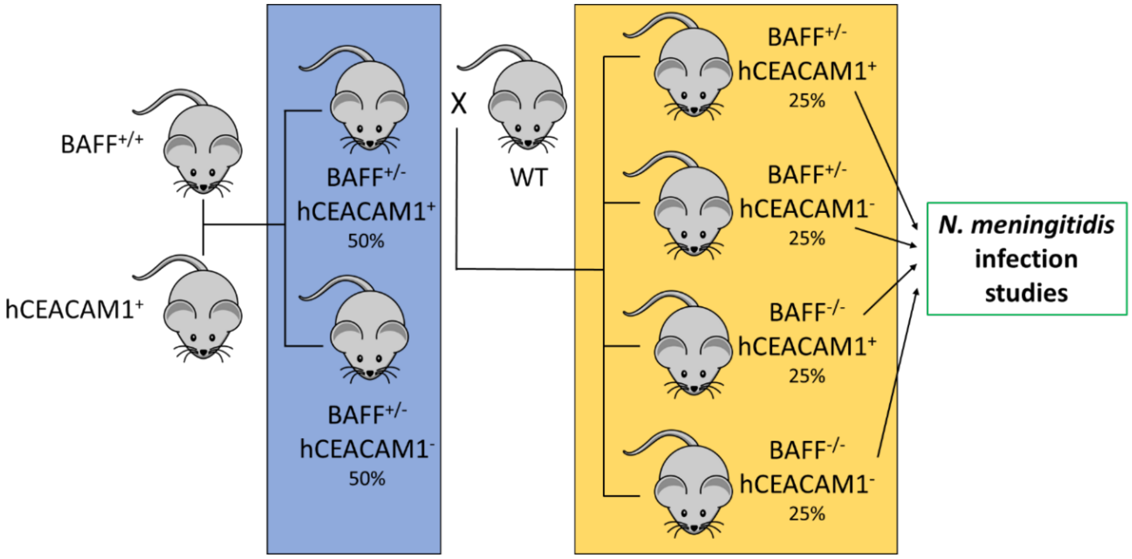

Supplement: Supplemental data [file jciinsight-7-141289-s114.pdf]
